# Head and Neck Tumor Segmentation on MRIs with Fast and Resource-Efficient Staged nnU-Nets

**DOI:** 10.1007/978-3-031-83274-1_6

**Published:** 2025-03-03

**Authors:** Elias Tappeiner, Christian Gapp, Martin Welk, Rainer Schubert

**Affiliations:** UMIT Tirol – Private University for Health Sciences and Health Technology, Eduard-Wallnöfer-Zentrum 1, Hall in Tirol 6060, Austria

**Keywords:** staged nnU-Net, MRI, Head and Neck Tumor, segmentation

## Abstract

MRI-guided radiotherapy (RT) planning offers key advantages over conventional CT-based methods, including superior soft tissue contrast and the potential for daily adaptive RT due to the reduction of the radiation burden. In the Head and Neck (HN) region labor-intensive and time-consuming tumor segmentation still limits full utilization of MRI-guided adaptive RT. The HN Tumor Segmentation for MR-Guided Applications 2024 challenge (HNTS-MRG) aims to improve automatic tumor segmentation on MRI images by providing a dataset with reference annotations for the tasks of pre-RT and mid-RT planning.

In this work, we present our approach for the HNTS-MRG challenge. Based on the insights of a thorough literature review we implemented a fast and resource-efficient two-stage segmentation method using the nnU-Net architecture with residual encoders as a backbone. In our two-stage approach we use the segmentation results of a first training round to guide the sampling process for a second refinement stage. For the pre-RT task, we achieved competitive results using only the first-stage nnU-Net. For the mid-RT task, we could significantly increase the segmentation performance of the basic first stage nnU-Net by utilizing the prior knowledge of the pre-RT plan as an additional input for the second stage refinement network. As team alpinists we achieved an aggregated Dice Coefficient of 80.97 for the pre-RT and 69.84 for the mid-RT task on the online test set of the challenge. Our code and trained model weights for the two-stage nnU-Net approach with residual encoders are available at https://github.com/elitap/hntsmrg24.

## 1 Introduction

Tumors in the Head and Neck (HN) region are primarily treated with radio-therapy (RT) [1]. MRI-guided RT planning offers several advantages over traditional CT-based approaches, particularly in terms of superior soft tissue contrast and the potential for daily adaptive RT due to the reduction of radiation side effects [21]. Intra-therapy MRI imaging allows for more precise targeting of tumors while sparing surrounding healthy tissues, which is crucial given the complex anatomy and critical structures in the HN region [11]. Despite these advances, the process of manually segmenting HN tumors on MRI scans remains labor-intensive and time-consuming, limiting the feasibility of fully utilizing MRI-guided adaptive RT in clinical practice [14]. The HNTS for MR-Guided Applications 2024 challenge (HNTS-MRG) aims to address this gap by providing a comprehensive dataset and a competitive platform for the development and benchmarking of automated HN tumor segmentation algorithms.

Participants in the HNTS-MRG 2024 challenge are tasked with developing algorithms to segment primary gross tumor volumes (GTVp) and metastatic lymph nodes (GTVn) on MRI scans, based on a training set of 150 patients. The algorithms are evaluated over the Grand Challenge platform^1^ on a hidden test set of 50 patients. The challenge is divided into two tasks. The first is focusing on pre-radiotherapy (pre-RT) planning and the second on mid-radiotherapy (mid-RT) planning. Notably, the second task encourages the exploration of data from prior timepoints to enhance segmentation performance.

Within this work, we present our approach to the HNTS-MRG 2024 challenge, which is based on the nnU-Net architecture with residual encoders of Isensee et al. [13]. Given the scarcity of high-end DL hardware since the rise of large language models in 2022 and the environmental impact of training these models [19], our primary focus is on the efficient use of computational resources while striving to achieve competitive results. Based on the hidden test data set of the challenge, we achieved an aggregated Dice Similarity Coefficient (aggDSC) of 80.97 for the pre-RT task using an nnU-Net with residual encoders and a target GPU training memory size of 24GB. For the mid-RT task we achieved an aggDSC of 69.84 by including the registered images of the pre-RT as additional input channels and applying a sampling strategy based on the available pre-RT segmentation mask. Being mindful and efficient in the use of computational resources our results show that convolutional neural networks (CNNs), especially the nnU-Net architecture, are still very well-suited for the given complex medical image segmentation using the given (relatively) small-sized datasets. For the mid-RT task, the incorporation of prior timepoint data significantly improved the training and inference times as well as the segmentation performance.

## 2 Dataset

The challenge dataset comprises 200 anonymized cases of patients with histologically confirmed HN cancer, primarily oropharyngeal cancer, treated with RT at the University of Texas MD Anderson Cancer Center. The dataset is split into 150 training cases, which are publicly available [28], and 50 hidden cases used for the online evaluation phase of the challenge. The dataset includes T2-weighted MRI scans captured pre-RT (1–3 weeks before treatment) for Task 1 of the challenge and mid-RT scans (2–4 weeks into treatment) for Task 2 of the challenge. Along with the MRI images, both tasks include corresponding GTVp and GTVn segmentations. Task 2 additionally includes elastically registered versions of corresponding pre-RT MRI scans and their pre-RT tumor segmentations. The segmentation of GTVs was performed by multiple expert observers and combined using the STAPLE algorithm [30] to produce consensus ground truth segmentations, with final validation by experienced radiation oncology faculty. The dataset is designed to support the training of machine learning models, and both training and test sets are structured to reflect real-world clinical cases. Ethical approval for the use of this data was granted by the MD Anderson Cancer Center’s Institutional Review Board. The hidden test set’s ground truth segmentations are not available until the end of the challenge.

## 3 Related Work

Most similar works with interesting insights for our participation in the HNTS-MRG 2024 challenge are the review papers of similar challenges such as the HEad and neCK TumOR (HECKTOR) segmentation in PET/CT challenge 2020 [2], 2021 [3] and 2022 [4], the Segmentation of Organs-at-Risk and GTV of patients with nasopharyngeal carcinoma for Radiotherapy Planning (SegRap) challenge 2023 [16], and the Head and Neck Organ at Risk (OAR) MR and CT segmentation (HaN-Seg) challenge 2023 [20].

The HECKTOR 2020 challenge, was the first large HN tumor segmentation challenge. Participants were asked to segment the primary GTV in the oropharynx region based on 201 FDG-PET/CT images. The best-performing team submitted ensembles of 3D U-Nets [7], which were trained using a combination of the Dice loss [17] and the Focal loss [15]. The second-placed team used a combination of a U-Net trained with Dice [17] and Top-K loss [31] functions and refined the results with an active contour approach. The third place was achieved using a classifier to identify slices with GTVs and a 2D U-Net [22] for the segmentation. The following HECKTOR 2021 challenge was expanded by two additional tasks and provided a training dataset of 224 PET and CT images. For Task 1, automatic HN GTV segmentation, the first, second, fourth and fifth ranked teams participated with ensembled nnU-Net [12] variants. The second-placed team implemented a staged approach that combined an initial rough segmentation with bounding box localization, followed by refined segmentations using a U-Net variant. In the most recent HECKTOR 2022 challenge, Task 1 was expanded to include the automatic segmentation of both primary GTV (GTVp) and metastatic lymph nodes (GTVn). The organizers provided an extensive training dataset of 524 segmented PET and CT images for the task. The first-place team utilized ensembled SegResNets [18] with automated parameter selection. The second-placed participants, similarly to the previous year, adopted a tumor localization and fine grained segmentation within the found bounding boxes. The final fine segmentation was achieved with ensembled nnU-Net and nnFormer [33] variants within this refined region. The third-placed team opted for an off-the-shelf nnU-Net [12] with basic pre- and post-processing rules.

The objective of the SegRap challenge [16] was the segmentation of 42 OARs (Task 1) in the HN region as well as GTVp and GTVn of nasopharyngeal carcinomas (Task 2) based on a training set of 140 non-contrast (ncCT) and contrast-enhanced CT (ceCT) images. For Task 2, the first-place team focused on efficient cropping and intensity clamping based on the HU values of ceCT and ncCT volumes, followed by training an nnU-Net-based segmentation network [12]. The second-place team used the UniSeg model [32], a pre-trained nnU-Net, with a bespoke pre-processing and ensemble strategy. The third-placed team also employed HU-based cropping before training different parameterized U-Net models [7], which are combined with additional test-time augmentation during inference.

The most recent challenge, the HaN-Seg 2023, differs from previous challenges by focusing solely on the segmentation of (OARs) in the HN region based on 40 MRI and CT images. We participated as team eli1 and achieved the first place using rigid registration of the MRI and CT images, followed by an nnU-Net-based [12] segmentation with an increased patch size. The second place was achieved using a YOLOv7 model [29] for the detection of the OARs, followed by OAR segmentation within the detected organ locations with a MONAI-based implementation of the nnU-Net named DynUNet. The third place was achieved using a pre-trained variant of the nnU-Net, trained with the class-adaptive Dice loss function [26] on rigidly registered MRI and CT images.

Amongst the studied challenges, the nnU-Net architecture has been a popular choice for many participants often leading to top results. The nnU-Net architecture introduced by Isensee et al. in 2021 [12] is a flexible and efficient deep learning framework for medical image segmentation based on the U-Net architecture [22]. Using a combination of dataset-dependent heuristics to define elements such as the spacing, patch-size, batch-size and the detailed network architecture as well as fixed hyperparameters such as the optimizer, learning rate, scheduler and loss functions, the nnU-Net architecture is able to achieve state-of-the-art performance without the need of extensive hyperparameter tuning. In a recent update, Isensee et al. [13] extended the base nnU-Net architecture with residual [10] encoders and reevaluated its performance on six different medical image segmentation tasks against other CNN-, transformer- [27] and mamba-based [9] architectures. The results show that the nnU-Net architecture with residual encoders is able to achieve high-quality performance while being more memory and runtime efficient than the other architectures. Although the newly presented nnU-Net clearly outperforms the observed transformer and mamba architectures, it is slightly outperformed by another CNN-based method, the MedNext [23]. According to Isensee et al., the MedNeXt’s performance gains are mainly explained by a substantially increased training time and the target spacing selection. Concluding, the authors tie the dominance of CNNs to the evaluation setting of training methods from scratch on benchmarks with limited dataset sizes, a setting which we also face in the HNTS-MRG 2024 challenge.

Besides the nnU-Net as a common architecture choice, many challenge participants applied multi-staged approaches combining a localization and segmentation stage. In earlier work, we also investigated the usage of a two-staged training approach for the HN organ at risk segmentation [25]. To refine an initial rough U-Net-based segmentation mask we apply a second training stage that is guided by the initial rough segmentation mask. The second stage operates on a reduced search space and benefits from a reduced within-class imbalance [26], which lead to improvements of the final segmentation performance.

## 4 Methods

Based on previous segmentation challenges and the reevaluation of the nnU-Net by Isensee et al. [13], which demonstrated its qualitative performance and computational efficiency, our method combines our previous work [25] using a two-stage training approach with the latest nnU-Net variant featuring residual encoders, the nnU-Net ResEncM.

### 4.1 Pre-RT Task

For the pre-RT Task, we first train an nnU-Net ResEncM with a GPU memory target of 24GB to utilize the full VRAM of the Nvidia Titan RTX available for training. Based on the dataset fingerprint of the challenge training dataset, the 3d fullres variant of the nnU-Net ResEncM is configured by the nnU-Net framework to have seven encoder/decoder stages, with a total of only 141 million trainable parameters. The network is trained with a dataset spacing of 0.5 *×* 0.5 *×* 1.2 mm, a patch size of 320 *×* 256 *×* 64, and a batch size of 2. By default, the optimizer used within the nnU-Net framework is Stochastic Gradient Descent with Nesterov momentum [6], trained with an initial learning rate of 0.01 and polynomial learning rate decay. The loss function of the nnU-Net is set as a combination of the Dice loss [17] and the Cross-Entropy loss, trained with deep supervision. During training the input data is augmented with the default nnU-Net augmentation techniques. Final segmentation results are obtained using a Gaussian-weighted sliding window approach with patch overlaps.

**Fig. 1.**
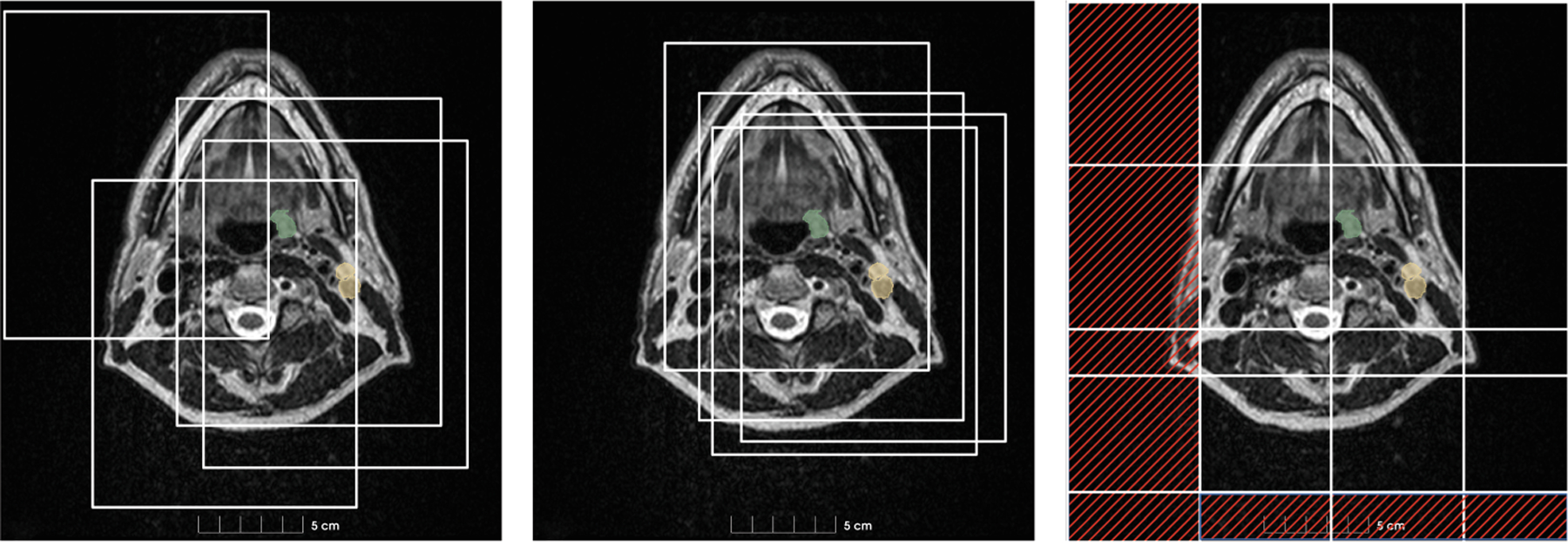
Left: Training process of the first stage networks using the default nnU-Net sampling strategy. Middle: Training process of the second stage networks with mask-based oversampling, using masks from the first stage (pre-RT Task) or the pre-RT reference segmentations (mid-RT Task). Right: Sliding window inference with 50% overlap; marked areas are not inferred for the mid-RT Task.

The second refinement stage is similar to our previous work [25], where a model is trained based on the segmentation output of the first stage. The first stage output is used as an additional input, guiding the patch sampling process during training by allowing only samples drawn from within the segmentation mask of the first stage. With the first stage providing candidate regions for the segmentation, the second stage can focus on refining the initial segmentation mask, as the patches are now likely to contain larger portions of the GTVp or GTVn than in the first stage. Compared to many staged methods applied in previous challenges, we do not use a separate localization network or bounding box detection to guide the segmentation process. This results in a straightforward approach that allows the reuse of the same network architecture in both stages. Notably, our method contrasts with the cascaded configuration of the nnU-Net, which uses the output of a first low-resolution training stage as an additional input channel for the second high-resolution training stage. Additionally, our patch sampling strategy differs from the default nnU-Net patching, which draws 33% of the training patches to include foreground label information based on the ground truth reference for training stability. Figure 1 (left and middle) illustrates the training process of the first and second stage networks.

### 4.2 Mid-RT Task

Unlike the pre-RT Task, where only a single MRI image is available, the mid-RT segmentation Task allows the utilization of the intra-therapy MRI, the registered pre-RT MRI, and the segmentation mask of the pre-RT GTVs. Assuming that the RT treatment is effective, reduced size GTVp and GTVn are expected to be in the same locations as found in the pre-RT MRI. Accordingly, for the mid-RT Task, we utilize the segmentation mask of the pre-RT phase as the additional input to guide the patch sampling process, requiring the training of only the second-stage nnU-Net ResEncM. In contrast to the pre-RT Task, where the first stage output is not guaranteed to be accurate, we also use the pre-RT segmentation mask for the sliding window inference, by again only considering patches drawn from within the segmentation mask as shown in Fig. 1 (right).

For the training process, due to limited access to large amounts of high-end DL hardware and the narrow time frame between dataset release and challenge submission deadline, we randomly split the training data for the pre-RT and mid-RT tasks into a single training and validation set of 120 and 30 patients, respectively. Both the first and second stages of the nnU-Nets are trained as defined by the nnU-Net framework for 1000 epochs, with each epoch consisting of 250 training steps. The code of the nnU-Net with our implementation for the two-stage training as well as trained model weights are available at https://github.com/elitap/hntsmrg24

## 5 Results

Since the HECKTOR 2022 challenge, the results of the automated GTVp and GTVn segmentation are evaluated based on the aggregated Dice Similarity Coefficient (aggDSC) [5]. The final ranking of the challenge is based on the average aggDSC of the GTVp and GTVn. Unlike the per-patient DSC, the aggDSC is robust against single false negative or positive results.

**Fig. 2.**
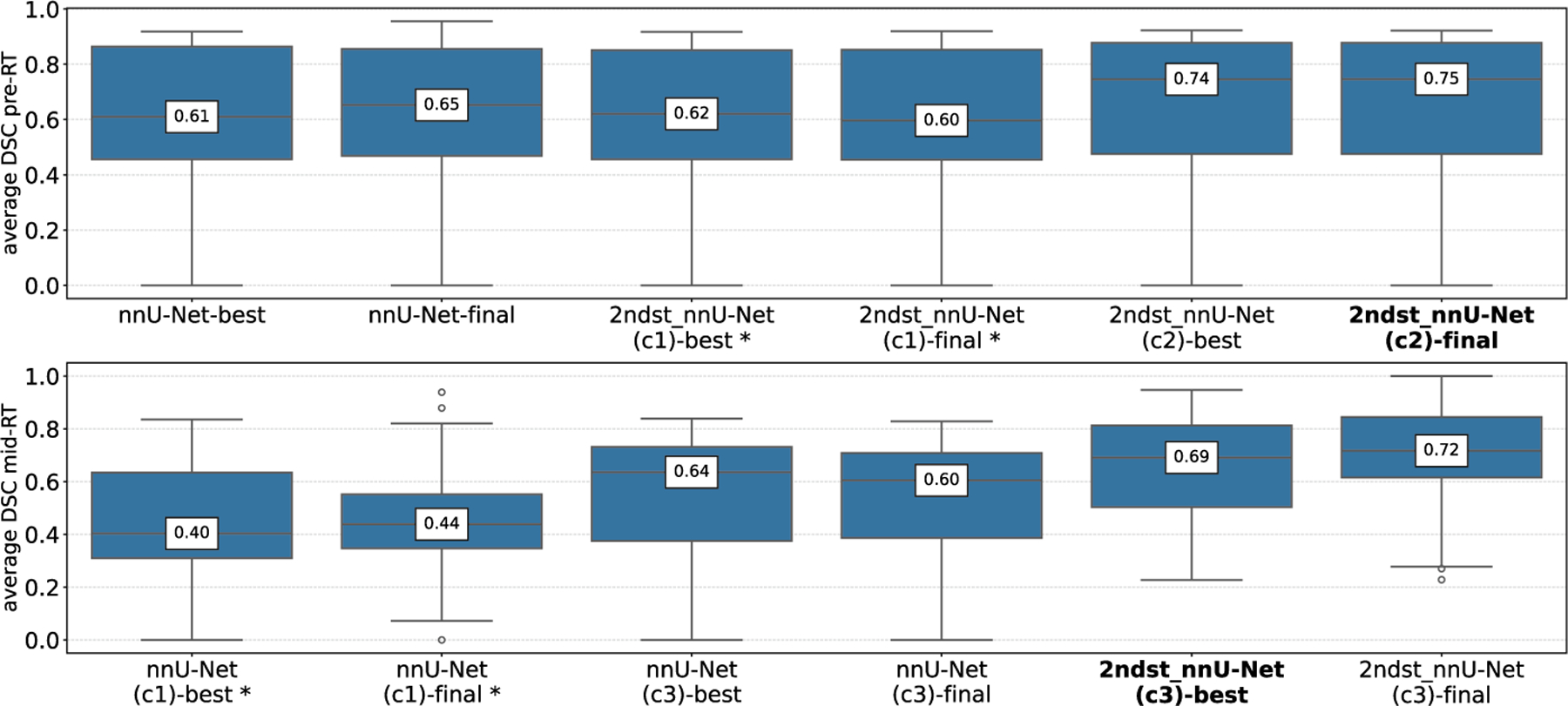
Individual average per-patient DSC results for the pre-RT task (top) and the mid-RT task (bottom). Configurations marked with a star (*) are significantly different from the best performing configuration marked in bold.

Based on our method, Table 1 presents the aggDSC results evaluated on our validation split of the dataset. To evaluate the impact of the second-stage training, Table 1 also contains the results of relevant ablation studies. For the pre-RT task, we also present the results of the first-stage nnU-Net, as well as two second-stage configurations. In the second stage nnU-Net (c1) configuration, we use the output of the first stage only for patch sampling, while for the second stage nnU-Net (c2) configuration, we use the output of the first stage as a full additional input channel for network training as well. For the mid-RT task, we train two first-stage configurations: one with the basic first-stage nnU-Net using the mid-RT MRI as the only input, and another with the registered pre-RT MRI and the pre-RT segmentation mask as additional input channels. Following the results from first-stage training, for the second stage the final configuration is trained using the mid-RT MRI, the registered pre-RT MRI and the pre-RT segmentation mask as input channels as well as the pre-RT mask for patch selection. Although the challenge is evaluated based on the aggDSC, individual per-patient measurements are of relevance for clinical applications. Accordingly, Fig. 2 also shows box plots of the average DSCs of each configuration for the pre-RT task (top) and the mid-RT task (bottom). Configurations marked with a star (*) significantly differ from the method with the best aggDSC marked in bold. The tests are based on a paired Wilcoxon signed-rank test with a significance level of 0.05.

Overall, we trained a total of six models for validation (including ablations) and an additional two models for the final submission. The training of a single nnU-Net in the given nnU-Net ResEncM configuration took approximately 83h under full utilization of the used Nvidia Titan RTX. We also adopt Code-Carbon [24] to track the computational efficiency of our approach, measuring an energy consumption of around 25 kWh, which corresponds to 2.8 kg CO_2_-equivalents, for the training of a single model. In summary, our entire challenge participation incurred marginal electricity costs of only 60 EUR for training in our region.

## 6 Discussion and Conclusion

The model selections for the final submission to the online evaluation of the challenge are based on the validation results of Table 1 and Fig. 2, as well as resource requirements. To utilize the whole training dataset for the final submission, we retrained the selected pre-RT and mid-RT models on the full 150-patient dataset.

For the pre-RT task, the best validation scores are achieved towards the end of the training process. Models with checkpoints at the best validation score achieve similar validation results as the fully trained models, except for the two-staged nnU-Net (c1) configuration, where we observed the appearance of islands in the segmentation masks outside the regions of the first stage mask. Due to our introduced sampling process, the network is expected to always contain foreground regions in the patches, and accordingly introduces the islands during inference. The problem is solved in the two-staged nnU-Net (c2) configuration, where the first stage output is used as an additional input channel, allowing the network to learn where to find the GTVp and GTVn segments. Ultimately, the second stage of the nnU-Net (c2) outperforms the first-stage nnU-Net baseline in terms of the aggDSC. However, our statistical analysis on the individual per-patient DSCs shows no significant difference from the nnU-Net ResEncM baseline configuration. Opting for resource efficiency, we submitted the basic first-stage nnU-Net configuration for the pre-RT task, achieving a final aggDSC of 80.97 in the online evaluation of the pre-RT task of challenge.

For the mid-RT task, the best validation scores are reached much earlier in the training process, and the models with checkpoints at the best validation score outperform the models trained for the whole 1000 epochs in all three configurations according to the aggDSC results of Table 1. Although Table 1 indicates signs of overfitting, the per-patient DSCs in Fig. 2 do not support this observation. Overall, both the aggDSC and the patient-wise DSC evaluations clearly show that the incorporation of the pre-RT data as additional input channels into the basic nnU-Net configuration improves the segmentation performance over the baseline model. We did not find a significant difference between the nnU-Net (c3) and the second stage nnU-Net (c3) configuration, however, the second stage nnU-Net (c3) configuration outperforms the basic nnU-Net (c3) configuration in terms of aggDSC and per-patient DSCs. Accordingly, for the final submission, we selected the two-staged nnU-Net (c3) configuration, where, due to the utilization of the pre-RT input as the first-stage output, only a single second-stage network needs to be trained. Additionally, the second stage nnU-Net (c3) configuration is much faster during inference as the pre-RT mask is also used to guide the sliding window inference. Finally, following our experience in training CNN-based networks for 3D medical image segmentation, where we found that longer training generally still improves distance-based metrics such as the Hausdorff Distance [25,26] as well as the configuration’s strong per patient DSC, we also trained the final model for the mid-RT task for 1000 epochs. Ultimately, the submitted second stage nnU-Net (c3) model achieves an aggDSC of 69.84 in the mid-RT task of the HNTS-MRG 2024 challenge. Table 2 presents the aggDSC of the online evaluation of the hidden test set from the Grand Challenge platform.

In conclusion, our results show that the medium sized nnU-Net architecture with residual encoders and a parameter count of only 141 million is still a competitive choice for the given complex medical image segmentation tasks, especially when only limited amounts of training data is available. Our two-staged training approach, where the first stage output is used to guide the sampling process for the second stage, is a straightforward and efficient method to improve the segmentation performance, however we found that the performance gains do not outweigh the training of a second nnU-Net. Nonetheless, the incorporation of prior timepoint data as input to a second-stage only network significantly improves the training and inference times as well as the segmentation performance of the mid-RT task, which is the major contribution of our work. Overall we can highlight that it is possible to achieve competitive performance using current consumer hardware and inexpensive-to-train standalone networks.

A compelling continuation in the spirit of this work would be to investigate the potential of patch curriculum learning, recently introduced by Fischer et al. [8]. The method involves training a network using progressively larger image patches, which the authors demonstrated could substantially reduce the number of training epochs needed to attain competitive results. Integrating this approach with our two-stage training method could enhance segmentation performance by guiding the initial, smaller patches to focus on relevant image regions. The combination may further bridge the performance gap between the pre-RT and mid-RT tasks.

## Figures and Tables

**Table 1.** aggDSC results for the epoch with the best validation metric (approximated DSC) and the fully trained final model evaluated on the validation data split, for the configurations of the pre-RT and mid-RT tasks. The best performing average results of each task and their configuration are marked in bold.

		best			final		
		GTVp	GTVn	avg (epoch)	GTVp	GTVn	avg
nnU-Net	pre-RT	79.56	87.45	83.51 (874)	79.72	86.95	83.33
2ndst nnU-Net (c1)		78.25	86.36	82.31 (941)	76.69	76.81	76.75
**2ndst nnU-Net (c2)**		79.73	87.45	83.59 (909)	79.97	87.61	**83.79**
nnU-Net (c1)	mid-RT	44.47	74.17	59.32 (104)	29.91	79.84	54.87
nnU-Net (c3)		62.90	78.21	70.55 (702)	52.19	82.90	67.55
**2ndst nnU-Net (c3)**		59.24	85.95	**72.59** (283)	53.87	81.49	67.68

**Table 2.** Final aggDSC results based on the online test set evaluation for the pre-RT and the mid-RT tasks.

	Task	GTVp	GTVn	avg
nnU-Net-final	pre-RT	76.71	85.22	80.97
2ndst nnU-Net (c3)-final	mid-RT	53.86	85.82	69.84
